# Characteristics of circulating CD31^+^ cells from patients with coronary artery disease

**DOI:** 10.1111/jcmm.12370

**Published:** 2014-09-30

**Authors:** Moo Hyun Kim, Longzhe Guo, Han-Soo Kim, Sung-Whan Kim

**Affiliations:** a Department of Cardiology, College of Medicine, Dong-A UniversityBusan, Korea; b Regional Clinical Trial Center, Dong-A University HospitalBusan, Korea; c Innovative Cell & Gene Therapy Center, International St. Mary’s HospitalIncheon, Korea

**Keywords:** angina pectoris, angiogenesis effect, coronary artery disease, CD31 antigen, inflammation

## Abstract

Recently, we reported the properties of CD31-expressing cells in healthy individuals. However, the characteristics of CD31-expressing cells derived from coronary artery disease (CAD) patients remain unknown. This study aimed to investigate the relationship between circulating CD31^+^ cells and CAD as well as their biological characteristics. Analysis with flow cytometry revealed that CD31^+^ cells (C-CD31) from the peripheral blood (PB) of CAD patients exhibited low levels of T-cell marker and high levels of macrophage marker compared with the PB-CD31^+^ cells from healthy individuals (H-CD31). In addition, the expression levels of multiple pro-angiogenic and chemokine genes were significantly down-regulated in C-CD31. However, inflammatory gene *IL-1*α was highly up-regulated in C-CD31. Patients with unstable angina (UA) had significantly more CD31^+^ cells in the PB than healthy control group (*P* < 0.001). Moreover, there were significant correlations between the number of CD31^+^ cells and cardiovascular (CV) disease activity (*R* = 0.318, *P* = 0.006) and the number of diseased coronaries (*R* = 0.312, *P* = 0.005). For the diagnostic category of UA, the area under curve was 0.803 (*P* < 0.001). In conclusion, C-CD31 have impaired angiogenic potential and the number of circulating CD31^+^ cells were correlated with CV risk. These findings may contribute to the understanding of the pathogenesis of CAD.

## Introduction

Cardiovascular (CV) disease is the most common disease in Western World. Despite many efforts to develop therapies, still numerous individuals are affected yearly from coronary artery disease (CAD), congestive heart failure, artrial fibrillation and stroke 1. Recently, cell-based therapy using stem or progenitor cells has been recognized as a promising strategy for the damaged tissue regeneration 2,3. However, various characteristics of stem or progenitor cells derived from CAD patients have not been fully elucidated.

There is extensive evidence proving the role of inflammation in CAD. Systemic inflammation has been proposed to play a major role in the pathogenesis of atherosclerosis and myocardial infarction 4–6. Immune cells such as monocytes and lymphocytes are mainly implicated in this process. When recruited, these inflammatory cells became potential mediators that contribute to the development of vessel injury by releasing reactive oxygen species (ROS) or proteolytic enzymes 7.

Platelet/endothelial cell adhesion molecule 1 (PECAM-1; CD31) plays a crucial role in cell transmigration *via* the intercellular junctions of endothelial cells. CD31 is expressed in neutrophils, monocytes 8, natural killer cells 9, haematopoietic progenitor cells 10, T cells, B cells and certain subsets of lymphocytes. Recently, we reported about the characteristics of CD31-expressing cells in healthy individuals 11. However, the characteristics of CD31-expressing cells derived from CAD patients are yet undiscovered. In addition, whether the number of CD31-expressing cells correlates with CV risk is unknown. To clarify these questions, we performed this study.

## Materials and Methods

### Study participants

We studied a total of 73 participants, comprising 21 control patients and 52 patients with CAD. Healthy individuals with no evidence of CAD, metabolic or inflammatory diseases by history and laboratory tests were used as controls. SA was defined as effort-related angina, which is the presence of chest pain without any change in its clinical pattern during the preceding 2 months. Unstable angina (UA) was defined as chest pain with an altered frequency, such as *de novo*, crescendo angina or angina at rest. The extent of disease was determined by counting the number of coronary arteries affected. The inclusion criteria were CAD patients with SA and UA and an age between 30 and 80 years. Acute myocardial infarction patients were excluded. The exclusion criteria were clinical evidence for the presence of chronic renal failure (serum creatinine >1.4 mmol/l), inflammatory disease, autoimmune disease, anaemia (haemoglobin < 8.5 g/dl) and thrombocytopaenia (100,000/l). The clinical details of the patients are shown in Table 1. The patient population size was estimated based on previous reports that demonstrated an increased number of circulating endothelial progenitor cells in patients with CAD 12.

**Table 1:** Clinical characteristics of patients

	Co	SA	UA	*P*
*N*	21	17	35	
Age (year)	59 ± 13	66 ± 10	63 ± 8	ns
Males, *n* (%)	15 (71)	11 (64)	25 (71)	ns
Hypertension, *n* (%)	0	11 (64)	15 (42)	-
Smoking, *n* (%)	4 (19)	3 (17)	10 (28)	ns
Diabetes mellitus, *n* (%)	0	6 (35)	15 (42)	-
Family history, *n* (%)	0	3 (17)	3 (9)	-
Troponin T positive, *n* (%)	0	0	14 (40)	-
CAD (1/2/3-vessel disease), *n* (%)	0	11 (65)/4 (23)/2 (12)	11 (31)/12 (34)/12 (34)	-
Medication, *n* (%)				
ACE-inhibitor	0	3 (18)	6 (17)	-
ARB	0	3 (18)	13 (37)	-
Aspirin	0	12 (70)	27 (77)	-
Beta-blocker	0	1 (5)	5 (14)	-
Calcium-blocker	0	10 (58)	25 (71)	-
Clopidogrel	0	2 (11)	21 (60)	-
Diuretics	0	7 (41)	11 (31)	-
Nitrate	0	6 (35)	15 (43)	-
Statin	0	8 (47)	24 (68)	-

Co, control patients; SA, stable angina; UA, unstable angina; na, not significant.

### Ethics Statement

Ethics approval for this study was received from the Institutional Review Board of Dong-A University Medical Center, and written informed consent was obtained from all participants before performing this study. The experimentation conformed to the principles established in the Declaration of Helsinki.

### Matrigel tube formation assay

A Matrigel tube formation assay was performed to assess the capacity to form networks. Matrigel (Becton Dickinson, San Jose, CA, USA) was added to chamber slides. After 1 hr, 2 × 10^4^ cells were seeded to each slide with 500 μl EBM-2 media containing 2% FBS. To investigate the integration potential of cells to form vascular structures, 0.2 × 10^4^ Dil-labelled cells were co-cultured with 1.8 × 10^4^ HUVEC. Eight hours later, seven representative fields were measured and the average total tube length was compared using Image-Pro Plus® (MediaCybernetics).

### Chemotaxis assay

The chemotaxis assay was conducted using the Transwell system (0.4 μm pores; Corning Costar Transwell, Cambridge, MA, USA). Briefly, VEGF-A (R&D system, Minneapolis, MN, USA) at a concentration of 100 ng/ml was added to the lower chamber, and 1 × 10^6^/well cells of each group were seeded into the upper 6-well chamber in serum-free DMEM. The transwell systems were then incubated for 48 hrs at 37°C. The number of cells that migrated into the underside of the inserted membranes was measured using five random separate fields.

### Adhesion assay

The adhesion assay was performed by modifying a previously reported method 13. The cells (1 × 10^5^/well) were seeded on 6-well plates pre-coated with 20 g/well type I collagen (Sigma-Aldrich) in DMEM for 2 hrs at 37°C and 5% CO_2_. After 2 hrs, the cells were gently washed three times with PBS and counted for adherent cells.

### Apoptosis assay

We induced apoptosis by treating the cells with camptothecin (6 μM, Sigma-Aldrich) for 4 hrs. Apoptotic cells were measured using propidium iodide (PI) and an Annexin V-FITC binding assay kit II (BD PharMingen, San Diego, CA, USA), according to the manufacturer’s protocol. The apoptotic cells were analysed using a FACScan (Becton Dickinson).

### Primers

The primers used for qRT-PCR were human VEGF-A (Hs99999070_m1), ANG-1 (Hs00181613_m1), hepatocyte growth factor (HGF; Hs00300159_m1), fibroblast growth factor (FGF-2; Hs00266645_m1), AKT-1 (Hs00178289_m1), IGF-1 (Hs01547657_m1), epidermal growth factor (EGF; Hs01099999_m1), GCP-2 (Hs00237017), IL-8 (Hs00174103_m1), monocyte chemoattractant protein (MCP-1; Hs00234040_m1), stromal cell-derived factor-1α (SDF-1α; Hs00171022_m1), IL-1α (Hs00174092_m1), TNF-α (Hs00174128_m1) and GAPDH (Hs99999905_m1). The following paired RT-PCR primers were used: 5′-catggaaacgaagcaccatc/ttggtggctcaacgcttaac-3′ for CXCR1 (217 bp), 5′- aaggcagaagcaacccaaat/tcaaggttcgtccgtgttgt-3′ for CXCR2 (145 bp), 5′-tacgccttcgttggtgagag/gcttattttgggttggcctc-3′ for CCR1 (212 bp), 5′-gaaggtggagaagctccctg/ttctttagccatgtggcctg-3′ for CCR2 (206 bp), 5′-atgactgtgagcggagcaag/ggaatgggatgtatctgccc-3′ for CCR3 (192 bp), 5′-gcaacatgctggtcatcctc/caaacacagcatggacgaca-3′ for CCR5 (279 bp), 5′ tggggcagcccagaacatca/gccgcctgcttcacacctt-3′ for GAPDH (198 bp). All primers and probes were purchased from Applied Biosystems (Foster City, CA, USA).

### Quantitative reverse transcription-polymerase chain reaction (qRT-PCR) and RT-PCR analysis

qRT-PCR assays were conducted as previously reported 14,15. The total RNA was isolated from cells using RNA-stat reagent (Iso-Tex Diagnostics, Friendswood, TX, USA), according to the manufacturer’s instructions, before the extracted RNA was reverse-transcribed using Taqman Reverse Transcription Reagents (Applied Biosystems). The cDNA was synthesized using human-specific primers and Taq polymerase (iNtRON Biotechnology, Sungnam, Korea). The DNA levels were quantitatively assessed using an ABI PRISM 7000 Sequence Detection System (Applied Biosystems). qRT-PCR cycling conditions were 10 min. at 95°C, followed by 40 cycles of 15 sec. at 95°C and 1 min. at 60°C. After normalization to GAPDH, the relative expression levels of the target gene in the experimental samples were determined using the formula Rel Exp = 2^−ΔCT^ (fold difference), where ΔCt = (Ct of target gene) − (Ct of control gene, GAPDH). The number of PCR cycles was measured using Lightcycler 3.5 software (Roche Molecular Biochemicals, Indianapolis, IN, USA).

### Flow cytometry

CD31^+^ cells were isolated using magnetic LS columns (Miltenyi Biotec) and flow cytometric analysis was conducted as previously described 3,11. Antibody used included the following: CD3, CD11b, CD14, CD19, CD31, CD34, CD45, CD146 (BD Bioscience, San Jose, CA, USA), CD133 (AC133; Miltenyi Biotec) and KDR (R&D Systems, Minneapolis, MN, USA). Flow cytometric data were analysed with FlowJo (Tree Star, Inc., Ashland, OR, USA) using appropriate isotype control.

### Patient characteristics

A total of 73 participants were included in this study, and their baseline characteristics are summarized in Table 1.

### Risk factors for CAD

The risk factors for CAD were considered as follows: diabetes mellitus (defined by the use of insulin or an anti-diabetic drug), hypertension (defined by the use of hypotensive drugs or having a repetitive hypertensive blood pressure), smoking (more than 2 packs per year) and family history (documented evidence of CAD in a close relative). The score of risk factors was calculated using age >65, male, smoking, hypertension, diabetes and a family history for CAD 12.

### Sample size and power consideration

When a one-way anova has 80% power to detect at the significant level of 0.05 a difference in means characterized by a variance of means, Σ(μ_i_ − μ)^2^/3 of 16.80 (μ_1_ = 14.20; μ_2_ = 22.45; μ_3_ = 13.37) assuming that the common standard deviation is 8.00, the sample size in each of three groups is 14. Assuming that the drop-out rate is 10%, 16 per each group will be required (a total number of sample size is 48) 16.

### Statistical analysis

Statistical analysis was performed with the SPSS program package (SPSS version 12.0; SPSS, Chicago, IL, USA). The differences between the two groups were compared using Student’s *t*-test. The groups were compared using one-way anova testing. Where heterogeneity of variance was identified, the data were confirmed using the non-parametric Mann–Whitney *U*-test. Categorical variables were compared using Pearson’s chi-squared test. For the association of CV risk factors with disease parameters, *P*-values were corrected by ancova models. Correlations between the parameters were calculated using Spearman’s method. The area under the receiver operating characteristic (ROC) curve was used to determine the sensitivity and specificity of the number of CD31-expressing cells for diagnosing UA. We assessed the optimum ‘cut-off’ number of CD31-expressing cells. NQuery Advisor version 7.0 software was used for sample size calculation. A value of a two-tailed *P* < 0.05 was considered to be statistically significant for all tests. All data were demonstrated as the mean ± SD, unless stated.

## Results

### Characteristics of CD31^+^ cells

Previously, we demonstrated that magnetic-activated cell sorted circulating CD31^+^ cells expressed CD45 (99%), a pan-haematopoietic marker, CD14, a monocyte/macrophage marker, CD3, T-cell marker, CD19, B-cell marker and preferentially expressed endothelial markers, suggesting that CD31^+^ cells were heterogeneous haematopoietic cells 11.

**Figure 1:**
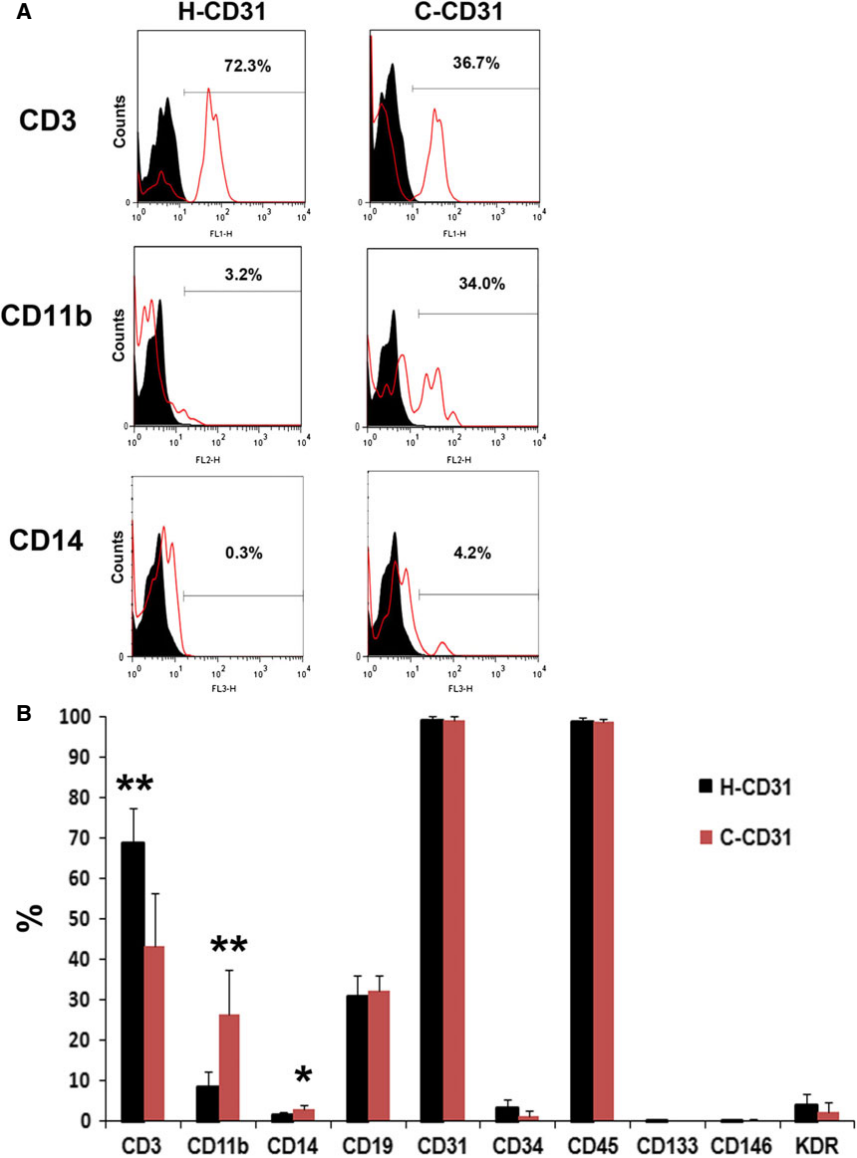
Haematopoietic characteristics of C-CD31. (**A**) Representative pictures of magnetic-activated cell sorted CD31^+^ cells analysed by flow cytometry. Black; isotype control, Red: specific antibody. (**B**) Quantification of the fluorescent-activated cell sorter data for C-CD31 and H-CD31. H-CD31 highly expressed T-cell marker (CD3) and C-CD31 markedly exhibited macrophage (CD11b) and monocyte marker (CD14). *n* = 21 per group. *P < 0.05, **P < 0.01.

To characterize H-CD31 and C-CD31, we isolated CD31^+^ cells using an immunomagnetic separation method and performed flow cytometric analysis. Surface marker analysis revealed that >99% of microbead-isolated CD31^+^ cells expressed CD31 and CD45. When comparing with these two groups, H-CD31 significantly expressed CD3, whereas C-CD31 markedly exhibited CD11b and CD14, underlining the differential composition of T cells and monocyte/macrophages in CD31^+^ cells (Fig. 1A and B).

### C-CD31 were impaired endothelial functions and survival capacities

**Figure 2:**
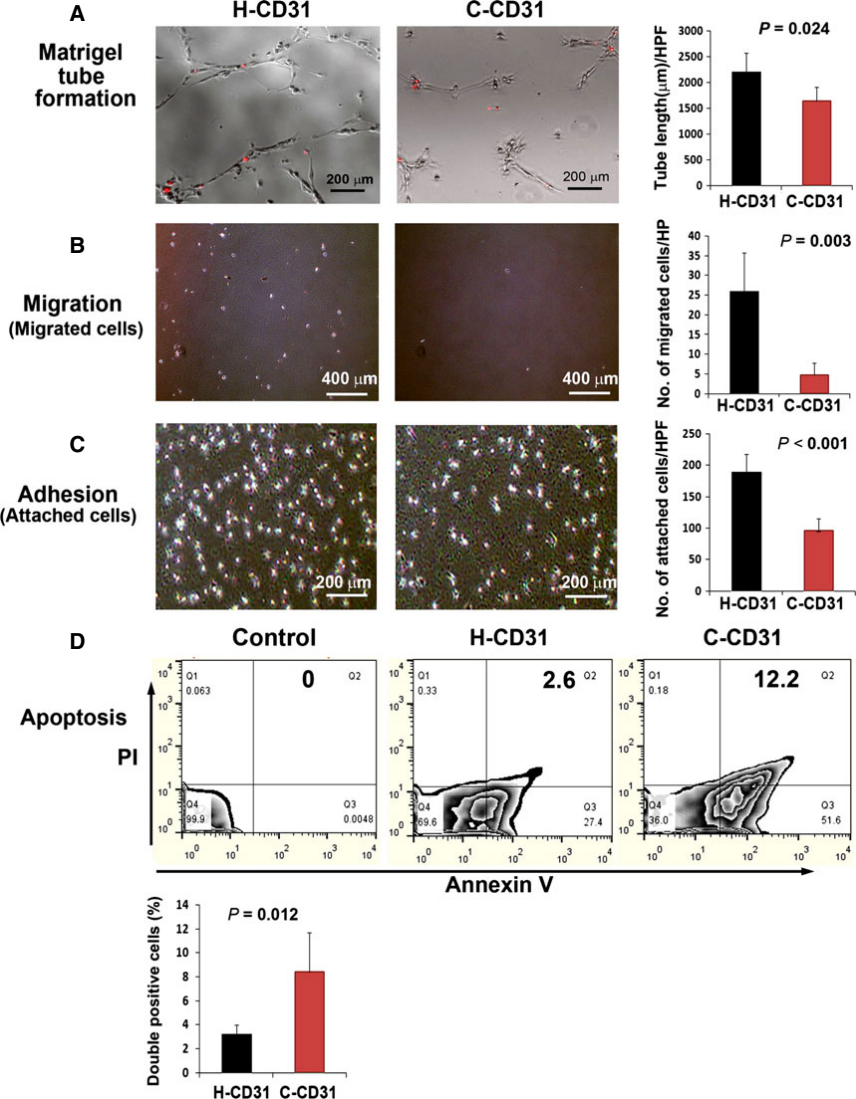
Evaluation of tube formation, migration, adhesion and anti-apoptotic potential. (**A**) Comparison of tube formation capability by co-culture with HUVEC. Tube length was measured 12 hrs after seeding of Dil-labelled cell in Matrigel coated plates. C-CD31 showed lower potential of tube formation. *n* = 5 per group. Chemotaxis (**B**) and cell adhesion (**C**) assays revealed that C-CD31 had lower migration and adhesion capacities than H-CD31 cells. *n* = 5 per group. (**D**) The apoptosis assay was analysed using a fluorescent-activated cell sorter (FACS). The level of both annexin V^+^ and propidium iodide (PI^+^) was significantly higher in C-CD31 than in H-CD31. *n* = 5 per group.

To determine whether C-CD31 were impaired during tube formation, cell migration, adhesion and anti-apoptosis, we isolated CD31^+^ cells using an immunomagnetic separation method and conducted Matrigel tube formation, chemotaxis, adhesion and apoptosis assays. First, we performed Matrigel tube formation assay by co-culture with HUVEC. The results showed that C-CD31 had lower tube formation capacity compare to H-CD31 (Fig. 2A). For the chemotaxis assay, we subjected cells in culture to a 100 ng/ml treatment with VEGF-A for 24 hrs. The exposure of C-CD31 for 24 hrs significantly decreased their migration compared with that observed for H-CD31 (*P* = 0.003) (Fig. 2B). Next, to examine the angiogenesis- and anti-apoptosis-associated function, we performed adhesion and apoptosis assays. The number of cells that adhered to the extracellular matrix protein collagen I was significantly smaller in the C-CD31 group than in the H-CD31 group (*P* < 0.001) (Fig. 2C). However, when apoptosis was induced, more apoptotic cells were discovered in the C-CD31 group than in the H-CD31 group (Fig. 2D).

### Characteristics of angiogenic, chemotactic and anti-apoptotic gene expression

**Figure 3:**
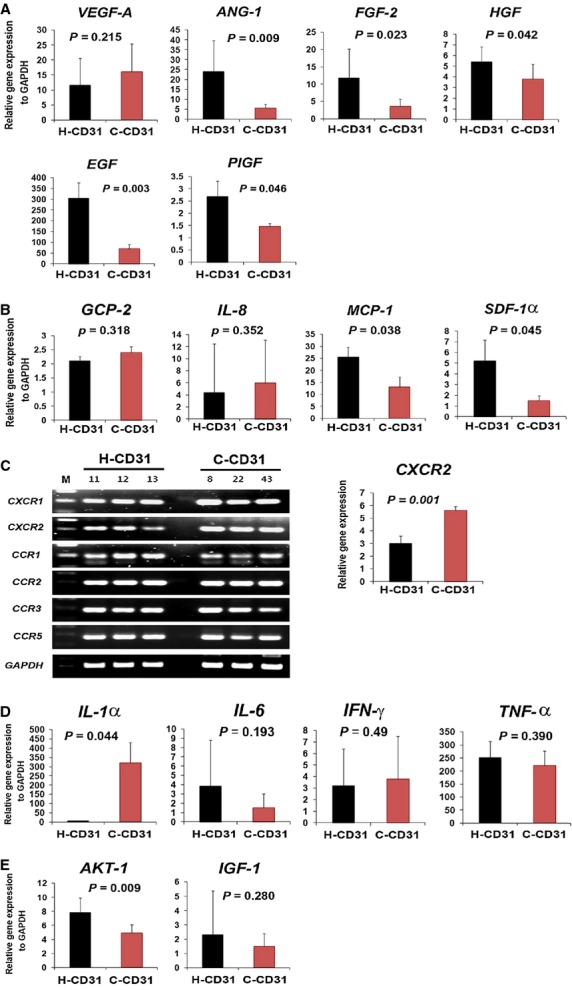
Evaluation of gene expression associated with the angiogenic, chemokine, anti-apoptotic and inflammatory properties. The expression patterns of multiple angiogenic (**A**), chemokine (**B**), chemokine receptor (**C**) anti-apoptotic (**D**) and inflammatory factors were measured in C-CD31 and H-CD31 using real-time reverse transcriptase polymerase chain reaction (RT-PCR). *n* = 6 per group.

To investigate the angiogenic potential of C-CD31, the expression patterns of multiple angiogenic factors were measured using real-time RT-PCR. Interestingly, multiple angiogenic factors, angiopoietin (*Ang*)-1, *FGF-2*, *HGF* and *EGF* were highly down-regulated in the C-CD31 group compared with the H-CD31 group (Fig. 3A).

Next, to identify chemokines and inflammatory characteristics, we measured the expression of chemokines and inflammatory factors using real-time RT-PCR. *MCP-1* and *SDF-1*α were significantly down-regulated in the C-CD31 group compared with the H-CD31 group (Fig. 3B). However, the chemokine receptor *CXCR2* and inflammatory factor IL*-1*α were significantly up-regulated in the C-CD31 group (*P* = 0.001, *P* = 0.044, respectively) (Fig. 3C and D). However, the representative anti-apoptosis-associated factor *AKT-1* was significantly down-regulated in the C-CD31 group (*P* = 0.009) (Fig. 3E).

### The number of CD31^+^ cells correlates with CAD

**Figure 4:**
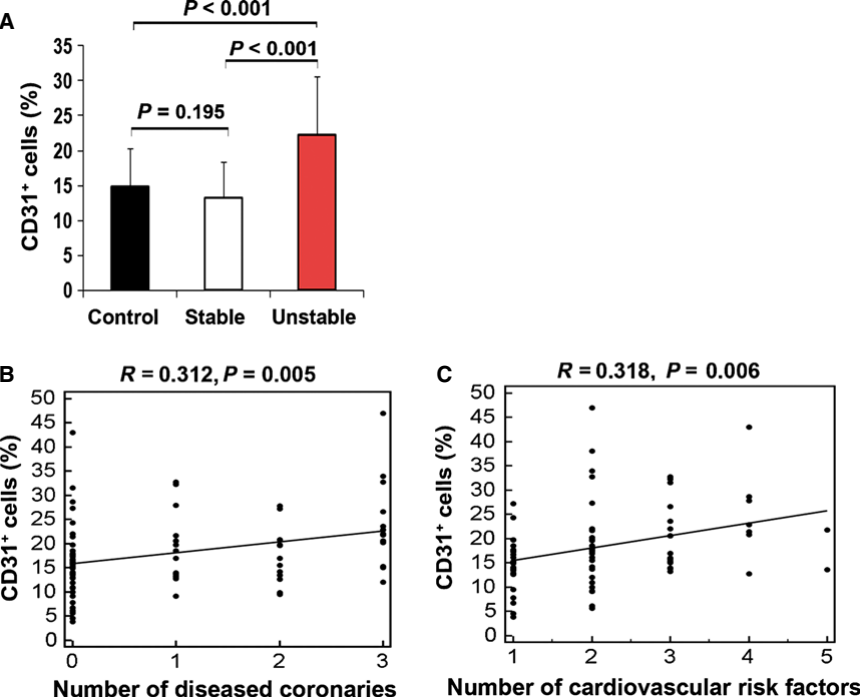
Proportion of circulating CD31^+^ cells and its relationship with diseased coronaries and cardiovascular risk. (**A**) The number of circulating CD31^+^ cells were compared in healthy controls and patients with SA or UA. Correlation of the number of circulating CD31^+^ cells with the number of diseased (stenosis >50%) coronaries (**B**) and individual cardiovascular risk factors (**C**) in healthy control and patients with SA or UA.

To determine the number of CD31-expressing cells in PB, the CD31^+^ cells were counted. One group of patients with UA, one group with SA and the healthy control group were prospectively included. Contrary to the previous studies of functional characterization, we included SA group. Because we speculated that the number of CD31-expressing cells might be related with disease severity. Interestingly, patients with UA had a significantly high number of CD31^+^ cells (22.2 ± 8.4) compared with the SA (13.4 ± 5.1, *P* < 0.001) and control group (14.2 ± 5.5, *P* < 0.001) (Fig. 4A). Moreover, the number of CD31^+^ cells correlated with the number of diseased coronary arteries (*R* = 0.312, *P* = 0.005) and CV risk factors (*R* = 0.318, *P* = 0.006) and (Fig. 4B and C).

### The number of CD31^+^ cells is a predictor of the CAD

**Figure 5:**
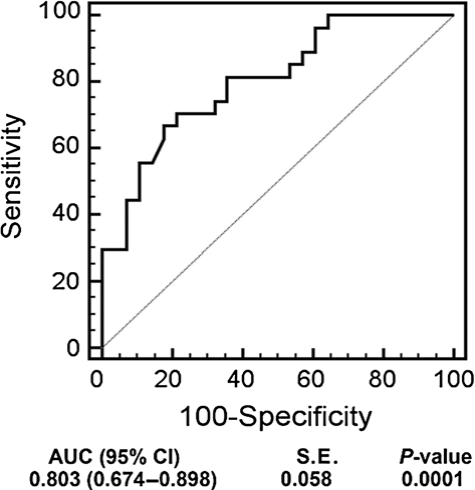
Receiver operating characteristic (ROC) curve assessing the number of CD31^+^ cells for diagnosing UA. Area under curve (AUC), which indicates the accuracy of the test. The cut-off value for level of CD31^+^ cells was 16.7% (95% CI 6.8–18.5).

The diagnostic value of the CD31^+^ cell count was confirmed by the mean area under the ROC curve (Fig. 5). For the diagnostic category of UA, the area under curve (AUC) was 0.803 (*P* = 0.005). A number of circulating CD31^+^ cells ≥18.5% was the best cut-off value for predicting UA (sensitivity = 70.4%; specificity = 78.6%).

### Inflammatory-associated genes are enriched in the CD31^+^ cell population

**Figure 6:**
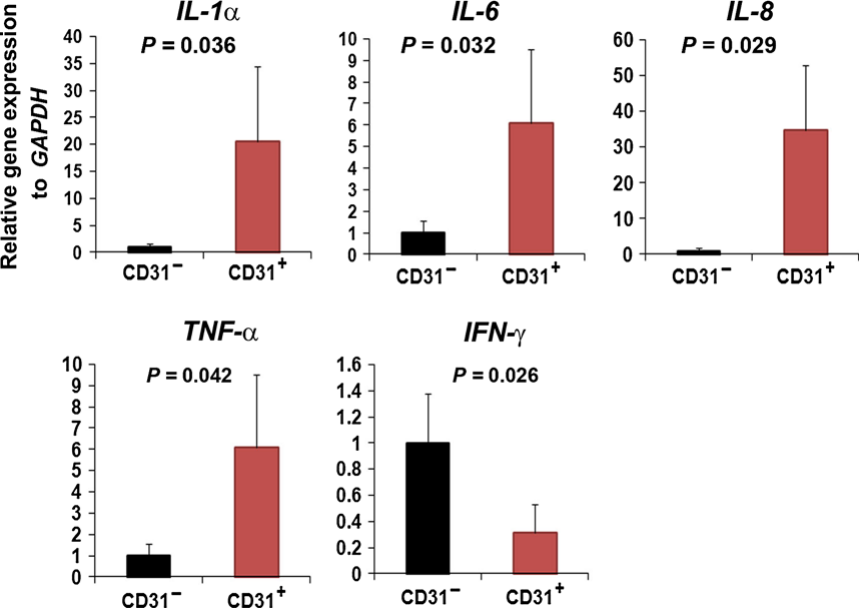
Real-time PCR analysis revealed the high inflammatory characteristics of human peripheral blood (PB)-derived CD31^+^ cells. The expressions of major inflammatory-associated genes were analysed using quantitative data of PB (**A**) and BM-derived CD31^+^ and CD31^−^ cells (**B**). *n* = 4 for each group.

To identify gene expression patterns between PB-derived CD31^+^ and CD31^−^ cells, we conducted a real-time RT-PCR analysis. The expression patterns of the inflammatory-related genes were compared. Quantitative data revealed that genes associated with the major inflammatory response or inflammatory signal pathway such as *IL-1*α*, IL-6, IL-8* and *TNF*-γ were significantly enriched in PB-derived CD31^+^ cells compared with CD31^−^ cells (Fig. 6). This result indicates that circulating CD31^+^ cells were mainly consistent with inflammatory-related cells.

## Discussion

This study was performed to investigate the relationship between circulating CD31^+^ cells and CAD as well as their biological characteristics. The important findings of present study are the following: (*i*) C-CD31 was composed of low levels of T cells and high levels of monocytes/macrophages; (*ii*) Endothelial functions and anti-apoptotic capacities of C-CD31 were impaired; (*iii*) the expression level of angiogenic and chemokine genes was down-regulated and inflammatory gene was up-regulated in C-CD31 and (*iv*) there was consistent association with a high number of CD31-expressing cells and escalating CV risk among a cohort of patients with UA. To the best of our knowledge, in this present study we were the first to report the properties of C-CD31 as well as the relationship between the number of circulating CD31-expressing cells and CV risk.

As CD31-cells are abundant in circulating blood, we speculate that these cells have advantages for uses in cell therapy and therefore further investigated their angiogenic properties 11. However, the nature of CD31-expressing cells in pathological environments is unknown. Interestingly, flow cytometric analysis showed that C-CD31 have low levels of T cells and high levels of monocyte/macrophages compared with H-CD31. These results suggest that C-CD31 is comprised of low numbers of angiogenic cells (CD3^+^CD31^+^ cells) and abundant inflammatory-associated cells.

The migratory role of cells is important for invading and integrating into ischaemic tissue and is strongly associated with physiological chemoattractants for cell recruitment because of the elevated level of *VEGF* and *SDF-1*α in hypoxic tissue 17,18. Therefore, we mimicked those conditions *in vitro* and tested the migration of cells. The chemotaxis assay indicated that C-CD31 were highly impaired in their migratory response. This phenomenon may be associated with a low expression of chemokines, *MCP-1* and *SDF-1*α in C-CD31 (Fig. 3B).

It was previously reported that BM-derived mononuclear cells (MNCs) from patients with chronic ischaemic heart disease exhibited a reduced neovascularization capacity 19,20. Therefore, we hypothesized that some MNCs may be responsible for the reduced angiogenic or neovascularization potential. As expected, a specific MNC population from CAD patients expressing CD31 expressed significantly low levels of pro-angiogenic factors, *ANG-1*, *FGF-2*, *HGF* and *EGF* (Fig. 3A). It has been noted that CD31 induces *AKT* phosphorylation and prevents apoptosis 21. In this study, we observed that CD31^+^ cells expressed *AKT-1*, a representative cell survival factor. *AKT-1* is significantly down-regulated in the CD31^+^ cells of CAD patients compared with those of healthy individuals, indicating that C-CD31 have a low cell survival capacity. Generally, vascular diseases elevate inflammatory cytokines and those cytokines activate ROS production in ischaemic tissue 22. It was reported that the inflammatory cytokine CRP inhibits angiogenesis and promotes the apoptosis of endothelial cells 17,18,23. Therefore, we speculate that an increased level of ROS or inflammatory cytokines may facilitate apoptosis and the dysfunction of CD31^+^ cells.

Another intriguing feature in this study was that C-CD31 significantly expressed the pro-inflammatory factor, *IL-1*α. *IL-1*α has been implicated in the formation of atherosclerotic plaques during the inflammatory process and acute coronary syndromes 24. *IL-1*α also plays important roles in reactive thrombocytosis related to inflammation. Because chemokines are important factors in inflammation and atherosclerosis progression, 25 we measured the properties of chemokines and chemokine receptors in CD31^+^ cells. Interestingly, a significantly elevated expression level of *CXCR2*, which is a major chemokine receptor of *CXCL1*, *CXCL2*, *CXCL3*, *CXCL5*, *CXCL6*, *CXCL7* and *CXCL8* 26, was detected in C-CD31. A recent report suggested that CXCR2 mediates the infiltration of inflammatory cells and myocardial protective role *via* chemotactic effects 27. Thus, *CXCR2* may play a key role in leucocyte recruitment during the inflammation and pathogenesis of myocardial infarction. Taken together, these results suggest that CD31^+^ cells may be a major source of inflammatory mediators, making an unfavourable contribution to the pathological process of CAD.

Recent reports revealed that CD3^+^/CD31^+^ T cells are related to acute coronary syndrome and cerebral small vessel disease in hypertensive patients 28. Consistent with these previous reports, we also discovered that an elevated number of CD31-expressing cells were detected in the patients with UA compared with the healthy control group. The exact mechanism responsible for the mobilization of the CD31-expressing subset in UA remains to be elucidated. However, our previous data demonstrated that CD31-expressing cells highly expressed chemokine receptors, including *CCR1*, *CCR2* and *CCR3* 11. Thus, those up-regulated chemokine-related factors may influence recruitment to damaged heart vessels. In addition, our real-time PCR data presented here demonstrate that both PB-derived CD31-expressing cells are highly enriched with inflammatory-associated genes (Fig. 6) and these results were consistent with our previous microarray data (data not shown) 11. In addition, there was a report that CD31 plays an important role in inflammation *via* its involvement in endothelial transmigration of leucocytes 27. However, it is not clear how UA causes the observed changes in numbers and characteristics of circulating CD 31^+^ cells.

In present study, our other findings indicate a correlation between the number of CD31^+^ cells and CV disease activity, which might predict adverse CV events. This important link also supports the hypothesis that the number of CD31^+^ cells is significantly related to the inflammatory process caused by endothelial cell damage. In summary, our data provided the evidence that C-CD31 are impaired in angiogenic properties and elevated numbers of CD31-expressing cells might be associated with inflammatory process in the pathogenesis of CAD. Therefore, monitoring the number of circulating CD31-expressing cells may be useful as an alternative predictor of CV risk for reducing the progression of CAD. However, the relative small sample size and the chosen cut-off value (50% stenosis) represent limitations of this study.

## Supporting Information

Table S1 Independent association of the level of the number of CD31^+^ cells with baseline characteristics.
