# Supplementary material for: Characteristics of circulating CD31+ cells from patients with coronary artery disease
Source: J Cell Mol Med. 2014 Sep 30;18(11):2321–30. doi: 10.1111/jcmm.12370 (PMC4224564; doi:10.1111/jcmm.12370)
Supplement: Table S1 — Independent association of the level of the number of CD31+ cells with baseline characteristics. [file jcmm0018-2321-sd1.doc]

**Supplemental Data**

**Title: Characteristics of circulating CD31^+^ cells from patients with coronary artery disease**

**Authors:** Sung-Whan Kim, PhD, Longzhe Guo, MD, Han-Soo Kim, PhD, Moo Hyun Kim, MD, PhD,

**Supplemental Table 1. Independent association of the level of the number of CD31^+^ cells with baseline characteristics**

**
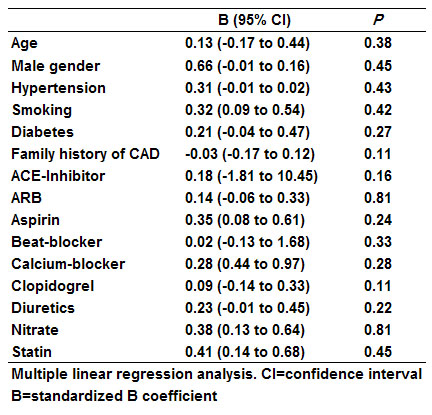
**
